# Cellulose-Based Polyurethane Foams of Low Flammability

**DOI:** 10.3390/polym16101438

**Published:** 2024-05-19

**Authors:** Marzena Szpiłyk, Renata Lubczak, Jacek Lubczak

**Affiliations:** Faculty of Chemistry, Rzeszów University of Technology, Al. Powstańców Warszawy 6, 35-959 Rzeszów, Polandrlubczak@prz.edu.pl (R.L.)

**Keywords:** (hydroxypropyl)cellulose, polyols, polyurethane foams, flame retardancy

## Abstract

Decreasing oil resources creates the need to search for raw materials in the biosphere, which can be converted into polyols suitable for obtaining polyurethane foams (PUF). One such low-cost and reproducible biopolymer is cellulose. There are not many examples of cellulose-derived polyols due to the sluggish reactivity of cellulose itself. Recently, cellulose and its hydroxypropyl derivatives were applied as source materials to obtain polyols, further converted into biodegradable rigid polyurethane foams (PUFs). Those PUFs were flammable. Here, we describe our efforts to modify such PUFs in order to decrease their flammability. We obtained an ester from diethylene glycol and phosphoric(III) acid and used it as a reactive flame retardant in the synthesis of polyol-containing hydroxypropyl derivative of cellulose. The cellulose-based polyol was characterized by infrared spectra (IR) and proton nuclear magnetic resonance (^1^H-NMR) methods. Its properties, such as density, viscosity, surface tension, and hydroxyl numbers, were determined. Melamine was also added to the foamed composition as an additive flame retardant, obtaining PUFs, which were characterized by apparent density, water uptake, dimension stability, heat conductance, compressive strength, and heat resistance at 150 and 175 °C. Obtained rigid PUFs were tested for flammability by determining oxygen index, horizontal flammability test, and calorimetric analysis. Obtained rigid PUFs showed improved flammability resistance in comparison with non-modified PUFs and classic PUFs.

## 1. Introduction

**Scheme 1:**
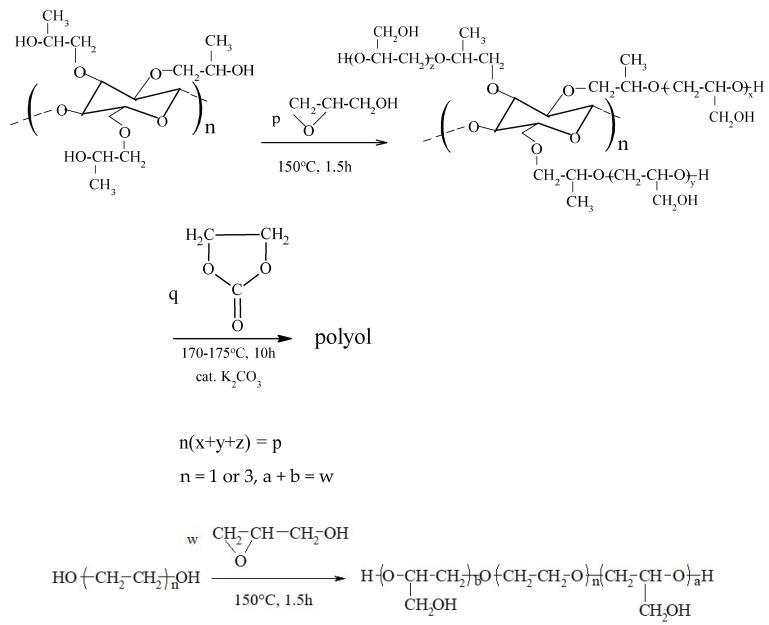
Obtaining polyols from HPC in the glycol medium.

Polyurethanes as foam materials are used in many areas of industry and daily life [1,2]. These materials are based on petrochemical substrates. The aspects of conscious environmental protection result in the search for solutions aimed at the production of plastics from natural, environmentally accepted, self-reproducing, and biodegradable sources [3], like cellulose. Cellulose was used as a filler in the fabrication of PUFs [4,5,6,7]. Cellulose is almost insoluble in water and organic solvents. Therefore, it is not readily convertible into polyols suitable to obtain PUFs. Recently, we have demonstrated that hydroxypropyl derivatives of cellulose (HPC) could be used as polyol substrates to obtain biodegradable, rigid PUFs [8,9,10]. Specifically, the HPC with three hydroxypropyl groups per glucose mere was reacted with glycidol (GL) in ethylene glycol (EG) or triethylene glycol (TEG). Obtained polyols were semi-solid resins, not miscible with isocyanates. In order to liquefy them, they were subjected to further hydroxyalkylation with ethylene carbonate [8] (Scheme 1).

**Scheme 2:**
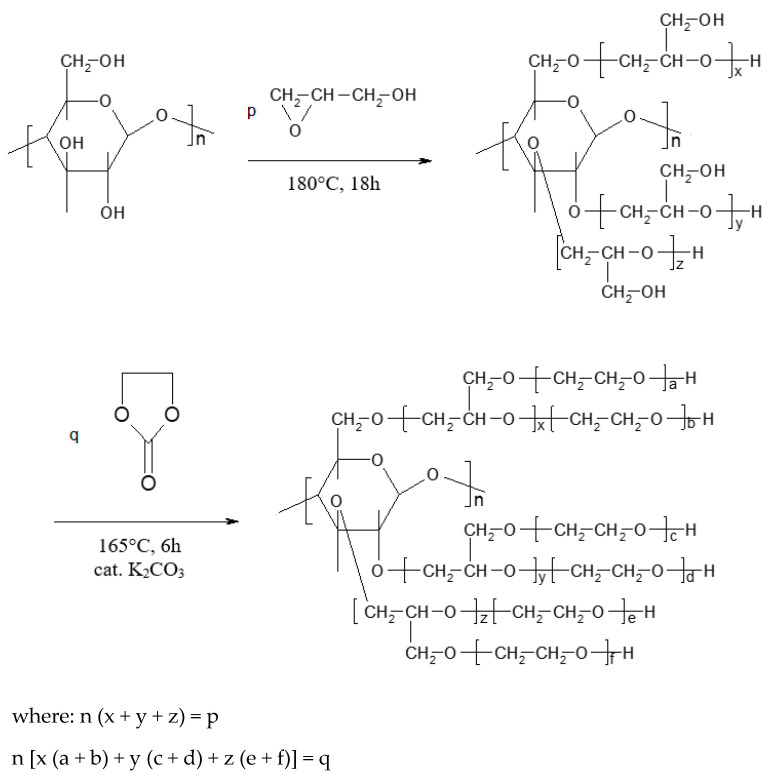
Obtaining polyols from cellulose.

Another approach to incorporating cellulose into PUFs was also applied; cellulose was preliminarily degraded by hydrolysis with 64% sulfuric (VI) acid at 45 °C for 2 h. Shortening of cellulose chains allowed us to use partially soluble oligomeric cellulose to react with GL and then with EC (Scheme 2) [9].

The least laborious method to obtain polyols from cellulose was based on preliminary swelling cellulose in water followed by hydroxyalkylation with GL and then with EC [10]. All three efforts led to polyols suitable for obtaining rigid PUFs with physical properties analogous to those of classic PUFs, except that they had elevated thermal resistance. They could withstand long-term thermal exposure at 150 °C; some of them could even resist 175 °C, while their compressive strength increased upon annealing. The advantageous feature of PUFs and polyols was readily biodegradation. The PUFs were 70–80% biodegradable within 28 days in laboratory conditions [10]. However, PUFs obtained from cellulose-based polyurethane foams are flammable.

Generally, flame retarding of PUFs can be achieved by incorporation of some elements like chlorine, bromine, nitrogen, or silicon into polyol or into PUFs [11,12]. Antipyrenes can result in a delay in the pyrolysis process, an increase in flame resistance, or even self-extinguishing of polymeric materials. Antipyrenes can be divided into two groups [13]: (i) reactive agents, whose atoms or groups are incorporated into the polymer structure during synthesis or crosslinking; and (ii) additive retardants, which do not react with polymer and are added in fabrication step. If phosphorus and nitrogen are incorporated in PUFs, they show synergy as flame retardants [14,15]. Nitrogen-containing melamine is often used as an effective additive retardant [16].

In the case of phosphorous retardants in PUFs, the mechanism of flame resistance leads to the formation of a glassy layer of phosphor oxide, which is unable to access oxygen and stop the formation of flammable and toxic gases [17,18,19]. The above-mentioned properties determined the choice of melamine and phosphoric acid ester as an additive and reactive flame retardant for a foamed composition containing polyol with the hydroxy-alkylated cellulose units. Here, the novelty is the synthesis of HPC-based polyol with incorporated phosphorus atoms, which resulted in a decrease in the flammability of obtained rigid polyurethane foams from the polyol. We obtained self-extinguishing rigid polyurethane foam with favorable performance properties.

## 2. Materials and Methods

### 2.1. Materials

The following materials were used: HPC average Mw~80,000, average Mn~10,000, powder, 20 mesh particle size, (99% purity, Merck, Darmstadt, Germany); GL (pure 98%, Sigma-Aldrich, Taufkirchen, Germany); ethylene carbonate (EC, pure ≥ 99%, Fluka, Buchs, Switzerland); potassium carbonate (anal. grade 100%, POCH, Gliwice, Poland); ethylene glycol (EG, pure, POCH, Gliwice, Poland); triethylene glycol (TEG, Merck, Darmstadt, Germany); diethylene glycol (DEG, p.a. Chempur, Piekary Śląskie, Poland); polymeric diphenylmethane-4,4′-diisocyanate (pMDI, Merck, Darmstadt, Germany; contains both bifunctional and trifunctional isocyanates); triethylamine (TEA, anal. Grade, Fluka, Buchs, Switzerland); surfactant Silicon L-6900 (pure, Momentive, Wilton, CT, USA); glycerol (GLYC, anal. grade 99.5–100%, POCH, Gliwice, Poland).

### 2.2. Synthesis of Polyol Using Phosphoric(III) Acid Esters

#### 2.2.1. Synthesis 1

EG (31.0 g, 0.50 mole), DEG (53.0 g, 0.50 mole) or TEG (75.0 g, 0.50 mol), and H_3_PO_3_ (20.5 g 0.25 mole) were placed in round bottom 250 cm^3^ flask equipped with Dean–Stark receiver and thermometer. Additionally, 110 cm^3^ of toluene was added in order to further azeotropic removal of water. The reaction mixture was heated to 112–115 °C. Water was distilled (9.0 cm^3^), and further excess of toluene was evaporated. The mixture was then cooled down, and HPC (13.56 g) and GL (30.7 g, 0.41 mole) were added. The mixture was heated at 190 °C until total GL was consumed (determined by the epoxide number of the sample; EN), and heating was continued for the next 15 min. After cooling, EC (111.8 g, 1.27 mole) and potassium carbonate catalyst (1.2 g) were added, and the mixture was heated at 170–180 °C for 24 h. The low-viscosity resins were obtained.

#### 2.2.2. Synthesis 2

Ester obtained from phosphoric(III) acid and from EG, DEG, or TEG (0.50 mole), as described above, was placed in a 250 cm^3^ flask equipped with a reflux condenser, mechanical stirrer, and thermometer. Then, HPC (11.7 g) was added, and the mixture was heated at 155 °C until HPC dissolved. Then, the mixture was cooled down to 50 °C, and GL (37.0 g, 0.50 mole) was added. The mixture was heated at 190 °C and/or cooled in case an increase in temperature was noticed due to exothermic reaction. Ultimately, the mixture was heated for an additional 15 min at 190 °C in order to increase the total consumption of GL (by EN determination). Then, the mixture was cooled down to 80 °C, and EC (130.0 g, 1.48 mole) and 1.0 g K_2_CO_3_ catalyst were added, and the mixture was heated at 180 °C until total consumption of EC.

### 2.3. Analytical Methods

The reaction with GL was monitored by epoxide number determination using hydrochloric acid in dioxane [20]. Specifically, 25 cm^3^ of hydrochloric acid solution in dioxane (1.6 cm^3^ in 100 cm^3^ dioxane) was added into a 0.5 g mass sample. Excess of HCl was then titrated with 0.2 M NaOH in methanol in the presence of *o*-cresol red as an indicator. The progress of the reaction of hydroksyalkylation with EC was monitored using the barium hydroxide method described in [21]. In particular, the samples of 0.1–0.5 g mass were treated with 2.5 cm^3^ 0.15 M Ba(OH)_2_ and then titrated with 0.1 M HCl in the presence of 0.2% thymoloftalein in alcohol. Finally, the hydroxyl number (HN) of polyols was determined by acylation with acetate anhydride in dimethylformamide. Thus, 1 g sample (with 0.0001 g precision) was heated with 20 cm^3^ acetylating mixture (acetic anhydride and dimethylformamide at 23:77 *v*:*v* ratio) for 1 h at 100 °C. Excess of anhydride was titrated with 1.5 M NaOH_aq_ in the presence of phenolphthalein. An acidic number of polyols was determined by alkalimetric titration described in [22]. Specifically, 0.1–0.2 g of polyol (with 0.0001 g precision) was placed in 20 cm^3^ water, and phenolophtalein (3 drops) was added and titrated with 0.1 M NaOH(aq) till the pink color of the indicator. The ^1^H-NMR spectra of reagents were recorded at 500 MHz BrukerUltraShield instrument in DMSO-d_6_ and D_2_O with hexamethyldisiloxane as the internal standard. IR was registered on the ALPHA FT-IR BRUKER spectrometer in KBr pellets or by attenuated total reflectance technique. The samples were scanned 40 times, in the range from 4000 to 450 cm^−1^ at 2 cm^−1^ resolution. Elemental HPC analysis was performed with EA 1108, Carlo Erba analyzer.

### 2.4. Physical Properties of Polyol

The density, viscosity, and surface tension of polyols were determined with a pycnometer, Höppler viscometer (type BHZ, prod. Prüfgeratewerk, Germany), and by the detaching ring method, respectively.

### 2.5. Polyurethane Foams

Foaming of polyol was performed at 500 cm^3^ cups at room temperature. The foams were prepared from 10 g of polyol, to which 0.26–0.27 g of surfactant (Silicon L-6900) and 0.46–0.89 g of TEA as catalyst and water (2%) as blowing agent were added. After homogenization, the pMDI was added in the amount of 18.0 g. The commercial isocyanate containing 30% of tri-functional isocyanates was used. The mixture was vigorously stirred until creaming began. The materials were then seasoned at room temperature for 3 days. The samples for further studies were cut from the obtained foam.

### 2.6. Properties of Foams

The apparent density [23], water absorption [24], dimensional stability in 150 °C temperature [25], thermal conductivity coefficient (IZOMET 2104, Bratislava, Slovakia), and compressive strength [26] of PUFs were measured. The apparent density of PUFs was calculated as the ratio of PUF mass to the measured volume of PUF sample in a cube of 50 mm edge length. Water volume absorption was measured on cubic samples of 30 mm edge lengths by full immersion of PUFs in water and mass measurement after 5 min, 3 h, and 24 h. Dimensional stability was tested on samples of 100 × 100 × 25 mm in size. The thermal conductivity coefficient was measured at 20 °C after 72 h of PUF conditioning. The needle was inserted 8 cm deep into a cylindrical PUF sample of 8 cm in diameter and 9 cm high. Compressive strength was determined using burden causing 10% compression of PUF height related to initial height (in accordance with the PUF growing direction). The thermal resistance of foams was determined both by static and dynamic methods. In the static method, the foams were heated at 150 and 175 °C for 30 days with continuous measurement of mass loss and determination of mechanical properties before and after heat exposure. The 100 × 100 × 100 mm cubic samples were used to determine the static thermal resistance and compressive strength. In the dynamic method, thermal analyses of foams were performed in a ceramic crucible at 20–600 °C temperature, with about 100 mg of sample, under an air atmosphere with 10 °C/min heating rate, recording time 60 min, DTA amplification 1/10, amplification DTG 1/5 with a Thermobalance TGA/DSC 1 derivatograph, METLER TOLEDO with STAR^e^ Software. DSC of PUFs was performed with scanning calorimeter METTLER TOLEDO 822^e^ using the following registration parameters: temperature range of −30–200 °C; heating rate of 10 deg/min; nitrogen atmosphere; sample mass 5–10 mg. The results were presented as thermal curves in terms of heat stream [W/g] versus temperature [deg].

Topological pictures of PUFs were recorded for cross-sections of PUF samples. The morphology of PUFs was investigated by an optical Panthera microscope (prod. Motic, Wetzlar, Germany) with 4 × 10 lenses and worked up with Motic Multi-Focus Professional 1.0 software, enabling merging and manipulation of images with adjustable lensing planes.

### 2.7. Flammability of Foams

The flammability of foams was determined by oxygen index and horizontal test according to norm [27] as follows: the foam samples (150 × 50 × 13 mm) were weighed, located on horizontal support (a wire net of 200 × 80 mm dimensions), and the line was marked at the distance of 25 mm from the edge. The sample was set on fire from the opposite edge using a Bunsen burner with a blue flame of 38 mm height for 60 s. Then, the burner was removed, and the time of free burning of foam reaching the marked line or cease of flame was measured by a stopwatch. After that, the samples were weighed again. The rate of burning was calculated according to the expression

(1) $$v=\frac{125}{t_{b}}$$

if the sample was burned totally or using the equation

(2) $$v=\frac{L_{e}}{t_{e}}$$

if the sample ceased burning, where

*L_e_*—the length of the burned fragment, measured as the difference of 150 minus the length of the unburned fragment (in mm). According to norms, if the burned fragment has a 125 mm length, the foam is considered flammable.

*t_b_*, *t_e_*—the time of propagations of flame measured at a distance between the starting mark and the end mark or as the time of flame ceases.

The mass loss Δ*m* after burning was calculated from the formula

(3) $$\Delta m=\frac{m_{o}-m}{m_{o}}.100\%$$

where *m_o_* and *m*—mean sample masses before and after burning, respectively.

Combustibility was determined by an oxygen index LOI instrument (Concept Equipment). The LOI value is the limited concentration of oxygen in a mixture of oxygen and nitrogen that is sufficient to sustain the combustion of the sample. The cuboidal PUF samples of 150 × 10 × 10 mm dimensions were placed in the middle of a vertical glass tube of 10 cm diameter and 70 cm length. Then, the quantitatively defined gaseous mixture of nitrogen and oxygen was passed through the tube. After 30 s of equilibration of the atmosphere inside the tube, the temperature was raised with a gas burner locally on top of the sample, and then the burner flame ceased. The burner was powered with propane, which gave 1860 °C flame temperature. The sample was considered combusted after the flame reached the line 8 cm below the top of the sample. The minimal oxygen concentration of oxygen in the gas mixture, which resulted in the continued flaming of the sample, was, thus, determined. The oxygen content in the gas mixture increased with 0.1% aliquot steps.

The flammability of foams was evaluated for samples 100 × 100 × 10 mm in size using a cone microcalorimeter, a product of FTT Ltd. (East Grinstead, UK), according to standard [28], by applying the heat flow 25 kW/m^2^ and the distance from ignition source 25 mm. During the tests, the time to ignite (TTI), total time of flaming (TTF), percentage mass loss (PLM), heat release rate (HRR), effective heat of combustion (EHC), and total heat release (THR) were recorded.

## 3. Results and Discussion

### 3.1. Synthesis of Polyol

We describe our efforts to modify previously obtained cellulose-based polyurethane foams [8] with flame retardants based on phosphorus and nitrogen derived from melamine. Due to the high viscosity of polyols (79,000–379,000 mPa s) obtained from HPC [8,9,10], an introduction of melamine was impossible. In order to obtain a lower-viscosity polyol containing phosphorus atoms in its structure, the first step was to esterify phosphoric(III) acid with ethylene glycol at a molar ratio of 1:2 using toluene as an azeotropic agent. The end-point of the reaction between acid and EG or TEG was determined by the mass of water as a side-product. TEG, similarly to EG, was used as a reaction medium during the preparation of polyols from HPC and GL [8]. The HPC, which was chosen for this process, had hydroxyl groups substituted with 3 hydroxypropyl residues per unit, as estimated based on the elemental composition of hydroxypropyl derivatives (Table 1). A good agreement was obtained between the calculated and determined compositions only for the tri-derivative, corresponding to HPC. Then, HPC was dissolved in the EG ester, and the mixture was hydroxyalkylated with GL and EC to obtain low-viscosity polyol with a high acidic number of 144 mg KOH/g. The use of this polyol to obtain PUFs needed a relatively substantial amount of catalyst (TEA) during the foaming process. Obtained PUF contained 2.6 mass% phosphorus and was self-extinguishing.

However, the foam was fragile. When TEG ester was used at the polyol synthetic step (AN = 51.5 mg KOH/g), the obtained PUF was rigid with small pores. The phosphorus % was 1.9; namely, it was too low to considerably reduce the flammability of the product.

**Scheme 3:**
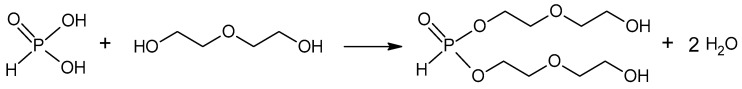
Preparation of DEG ester and phosphoric acid (III).

Finally, we succeeded in using the DEG ester of phosphoric(III) acid (Scheme 3).

AN for the polyol obtained with this ester was 60 mg KOH/g. Ultimately, the PUF obtained from the polyol was self-extinguishing (Table 2).

**Figure 1:**
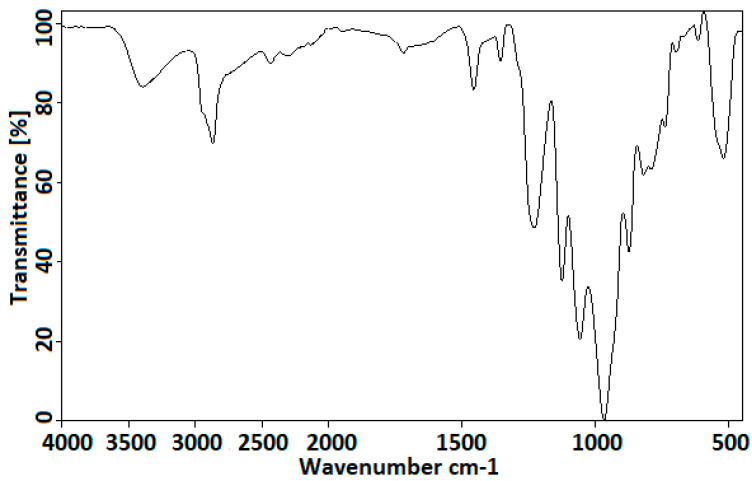
IR spectrum of phosphoric acid(III) and DEG ester.

**Figure 2:**
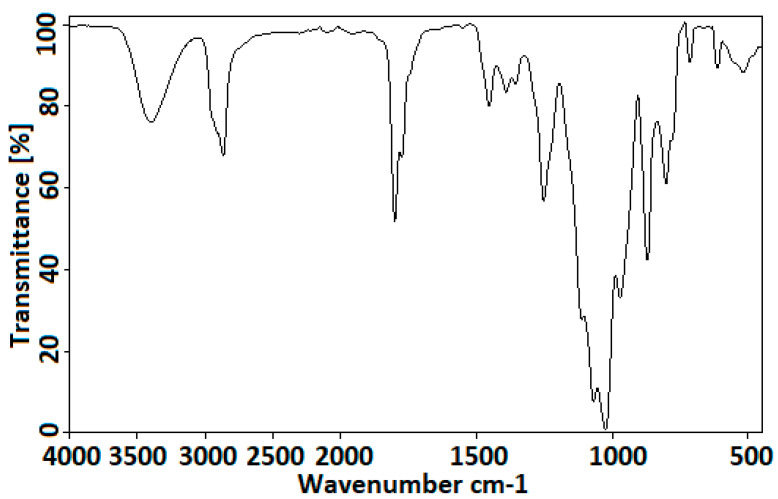
IR spectrum of polyol H_3_PO_3_-DEG + HPC-GL-EC.

Figure 1 shows the IR spectrum of DEG phosphate(III). The stretching and deformation vibrations of methylene groups were observed at 2868 cm^−1^ and 1456 cm^−1^, respectively. The O–H stretching and deformation vibration were found at 3395 and 1356 cm^−1^, while the C–OH stretching vibration was located at 1058 cm^−1^, characteristic of the primary hydroxyl group. They overlapped with stretching vibrations C–O–C. The characteristic P=O stretching vibrations were at 1230 and 1125 cm^−1^, and a strong P–O–C band was observed at 968 cm^−1^, while a small band at 2435 cm^−1^ was attributed to the P–H bond. The mentioned P=O and P–O–C bands were also observed in the IR spectrum of the obtained polyol (Figure 2). Ether bond bands at 1060 cm^−1^ were observed both in the spectrum of ester and in the products of HPC reaction with GL and EC. 

**Scheme 4:**
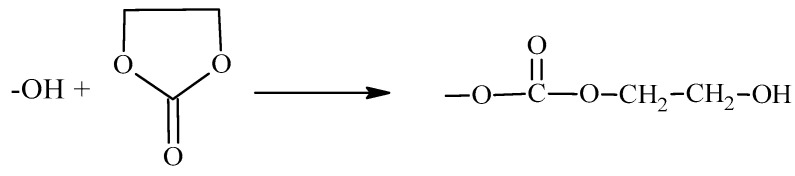
Formation of an ester bond in reaction with ethylene carbonate.

The stretching and deformation of O–H bands were found at 3450 cm^−1^ and 1353 cm^−1^, respectively. The band centered at 1750 cm^−1^ was attributed to polyol carbonyl stretching vibrations. The presence of this band suggested the incorporation of ester groups into the polyol structure (Scheme 4), while most ethylene carbonate was decomposed with the formation of hydroxyethyl groups and the elimination of carbon dioxide.

**Figure 3:**
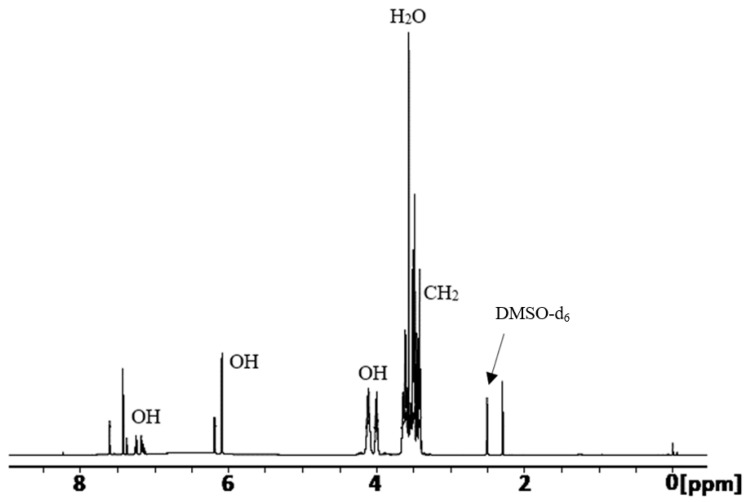
H-NMR spectrum of phosphoric acid(III) ester and DEG.

**Figure 4:**
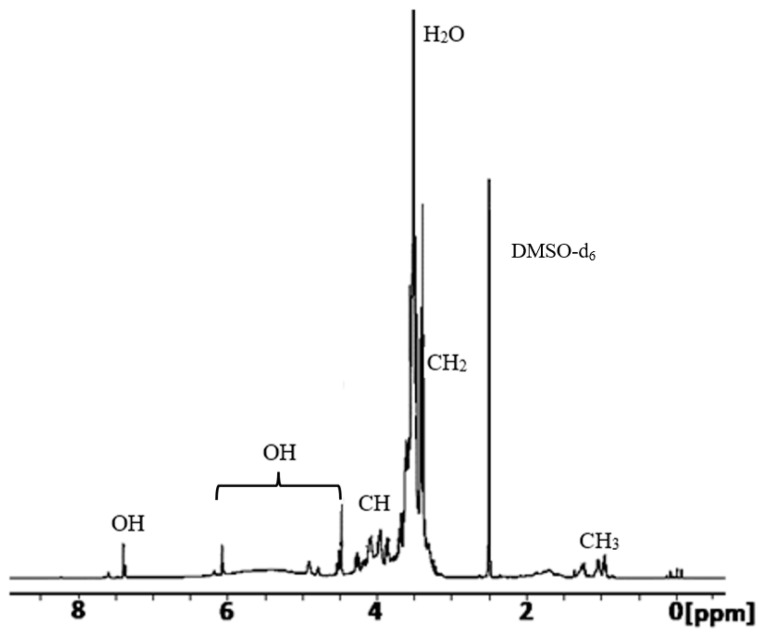
^1^H-NMR spectrum of polyol obtained from H_3_PO_3_-DEG -HPC-GL-EC.

The ^1^H-NMR spectrum of ester is simple (Figure 3) and composed of methylene protons signals within 3.4–3.6 ppm and hydroxyl proton resonances at 4.1 and 6.1 ppm and within the 7.2–7.6 ppm region, depending on various hydrogen bonds in different molecular setting. In the ^1^H-NMR spectrum of polyol (Figure 4), the resonances at the 3.4–3.6 ppm region overlap with resonances of methylene protons of hydroxyalkylated HPC, as well as an ester. The resonances within 1.0–1.2 ppm are obviously from methyl groups of HPC. The hydroxyl proton resonances were observed at 4.5, 5–6, and 7.4 ppm, which were identified by selective deuteration with D_2_O.

The physical properties of polyol obtained from GL and EC with the mixture of HPC and phosphoric(III) acid and DEG ester were determined. Thus, the density and surface tension of obtained polyol at 20 °C were 1.266 g/cm^3^ and 32.0 mN/m, respectively, while viscosity was 292 mPa s. The latter was many times lower in comparison with that of polyols obtained from HPC, GL, and EC in EG or TEG [8]. Also, the surface tension was 20 units lower than those of previously obtained polyols [8]. Thus, the polyol obtained here was suitable for further homogenization of foaming composition. Additionally, the hydroxyl number of this polyol was 400 mg KOH/g, indicating that the polyol was an appropriate candidate to obtain rigid PUF.

### 3.2. Polyurethane Foams

Foaming was performed on a laboratory scale (Table 3). The optimized water content in the foaming composition was 2% water in reaction to the mass of polyol. At higher water content (3%), the PUF was fragile, and below 2%, the PUF was under-foamed and had high apparent density. Surfactant content was optimized at 2.7 g, while the catalyst was at 7.8 g/100 polyol. At a lower amount of catalyst, the under-crosslinked PUF with a viscous surface was formed. On the other hand, fast growth and expansion of PUF were observed. Due to the low viscosity of polyol, the additive retardant melamine could be introduced into the foaming composition. 

**Figure 5:**
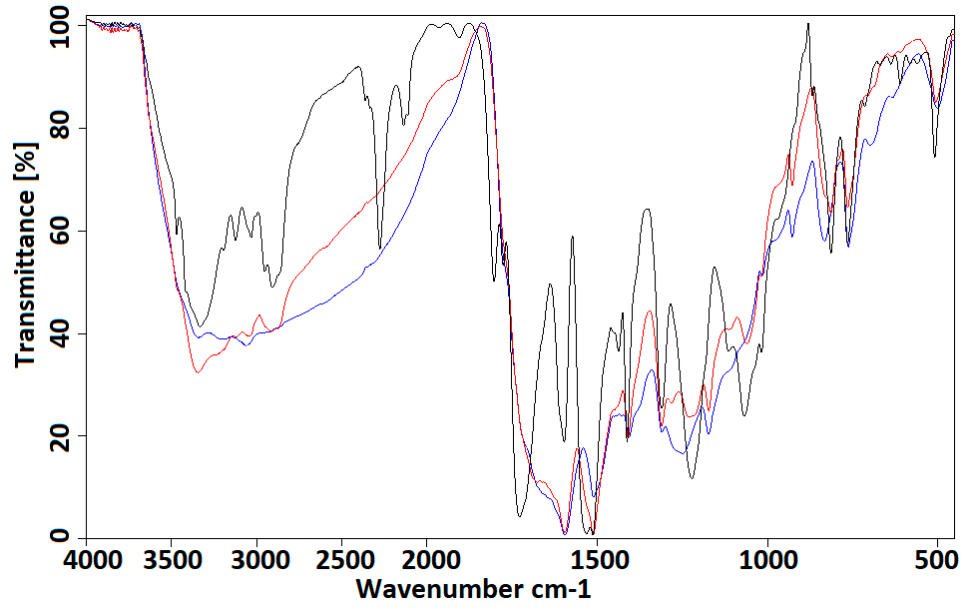
IR spectrum of unheated foam (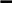) and foam exposed to temperatures 150 °C (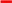) and 175 °C (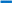).

The time was measured with a regular stopwatch, resulting in obtaining self-extinguishing PUFs. In that case, the cream time was 18 s, and rise and tack-free times were 9 s and 1 s, respectively. For comparison, the corresponding times in the case of HPC obtained before [8] were 45–61 and 35–37 s, while the tack-free time was 1 s. The shorter cream and rise times of the foam modified with flame retardants compared to the foams without flame retardant are due to the larger amount of catalyst used, which is needed to initiate the foaming process. We have observed a considerably higher isocyanate coefficient than that calculated with the assumption that one isocyanate group reacted with one hydroxyl group. This suggests the possibility of partial trimerization of isocyanates with the formation of isocyanurate rings in the foam. That process can occur due to the presence of large amounts of TEA in foaming compositions, which catalyzes trimerization [29]. In fact, the presence of characteristic bands in the IR spectrum of the PUF, namely, deformation band NH and C=O at 510 and 770 cm^−1^, indicates the presence of a perhydro-1,3,5-triazine ring in the product (Figure 5). The properties of obtained PUFs, together with the reference PUFs obtained from HPC without flame retardants, are shown in Table 4 and Table 5.

The obtained PUF shows good dimensional stability during thermal exposure at 150 °C, much better than the reference PUFs. Its maximum shrinkage after 40 h exposure was 1.76%, while it was 6% for reference PUFs (Table 4). The apparent density was 74 kg/m^3^, which was larger than reference PUFs, presumably due to the presence of melamine as flame retardant added into the foaming composition in the former. Water uptake of obtained PUF after 24 h exposure was 4.8%, probably due to the presence of melamine in the structure of the material, which enabled it to bind to water stronger than in reference PUF.

**Figure 6:**
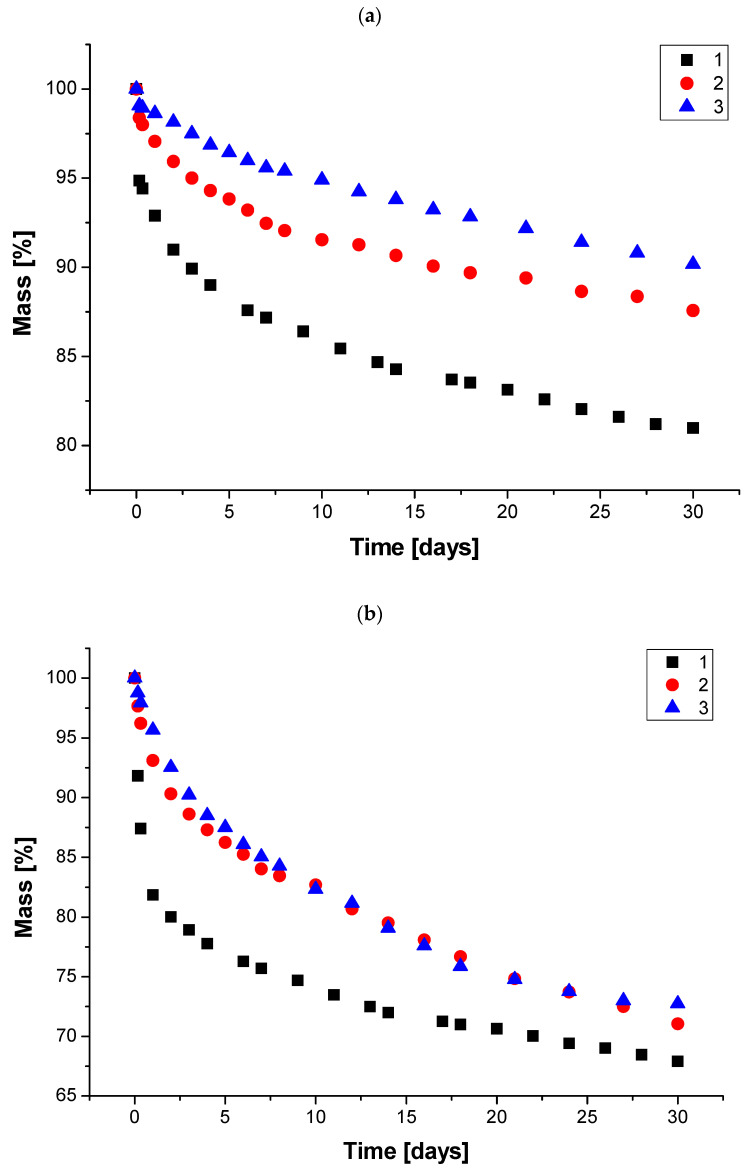
Thermal stability of the polyurethane foam as the mass loss after heating at 150 °C (**a**) and 175 °C (**b**) obtained from polyols H_3_PO_3_-DEG-HPC-GL-EC (1), HPC-GL-EG-EC (2), and HPC-TEG-GL-EC (3).

The heat conductance coefficient was 0.0361 W/mK and was slightly higher than the reference PUFs (Table 5). The thermal resistance of PUF was determined by thermal exposure of PUF at 150, 175, and 200 °C for 30 days with concomitant measuring mass loss and compressive strength before and after exposure. The rapid mass loss was observed on the first day of thermal exposure (Figure 6). PUFs with flame retardants had lower thermal resistance at 150 °C (higher weight loss) than reference PUFs. This might be due to the continuous removal of melamine by sublimation. At 200 °C, severe PUF deformation and mass loss were observed compared with the reference PUFs.

IR spectra of PUF before and after thermal exposure shed some light on the structural aspects of thermal exposure (Figure 5). In the IR spectrum of PUF that was not annealed, the band centered at 3350 cm^−1^ from N–H corresponds to the stretching vibrations involved in hydrogen bonding. The bands at 3000–2800 cm^−1^ were attributed to C–H stretching vibrations. Some unreacted isocyanate groups were discovered by the band at 2276 cm^−1^. Also, the band at 2136 cm^−1^ originated from carbodiimide groups. These bands disappeared upon thermal exposure at 150 °C, presumably due to oxidation and/or the reaction with free hydroxyl groups, leading to additional crosslinking. The band at 1727 cm^−1^ in the IR spectrum of starting PUF belongs to C=O of amide groups. Its intensity decreased upon thermal exposure, suggesting that the thermal decomposition of amide bonds took place. Another structural change in the PUF upon heating was identified by the presence of a growing band at 1610 cm^−1^, which corresponded to C=C stretching vibration. Such changes are related to the observed graphitization of samples upon prolonged heating. The band at 1250 cm^−1^ attributed to stretching vibration P=O of phosphoric(III) esters in PUF decreases upon heating of PUF.

The compressive strength of not-annealed PUF is 0.237 MPa and increases after thermal exposure of PUF to 150 °C, probably due to additional crosslinking of PUF. This supposition was confirmed by the disappearance of the N=C=O band at 2276 cm^−1^ in the IR spectrum of the annealed PUF, formerly present in the IR spectrum of the not-annealed one. At a higher temperature (175 °C), the compressive strength decreases upon degradation (Table 5). The PUF modified with flame retardants has lower compressive strength than reference PUFs. The presence of melamine in PUF resulted in a decrease in compressive strength, probably due to melamine sublimation of the sample.

**Figure 7:**
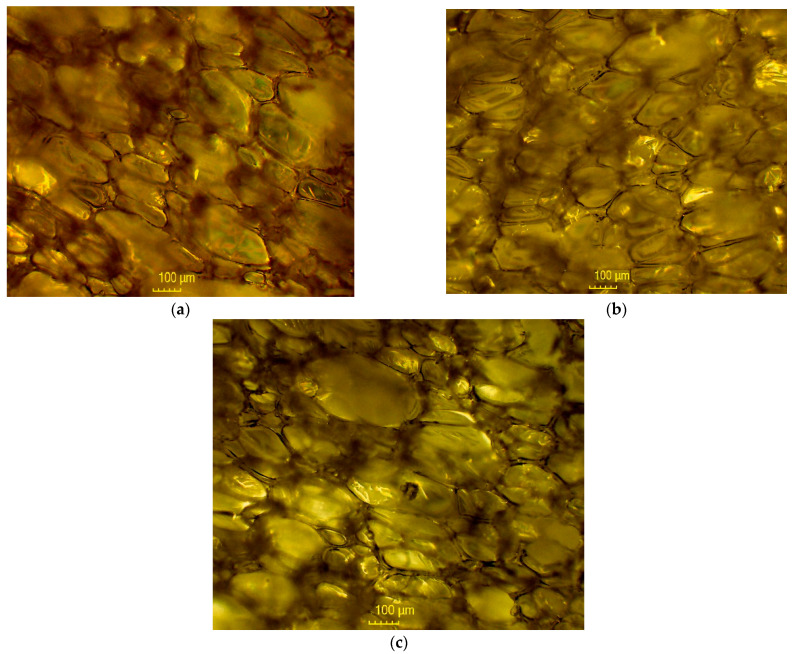
Optical microscopy images of foam obtained from polyols H_3_PO_3_-DEG-HPC-GL-EC (**a**), HPC-GL-EG-EC (**b**), and HPC-GL-TEG-EC (**c**).

**Figure 8:**
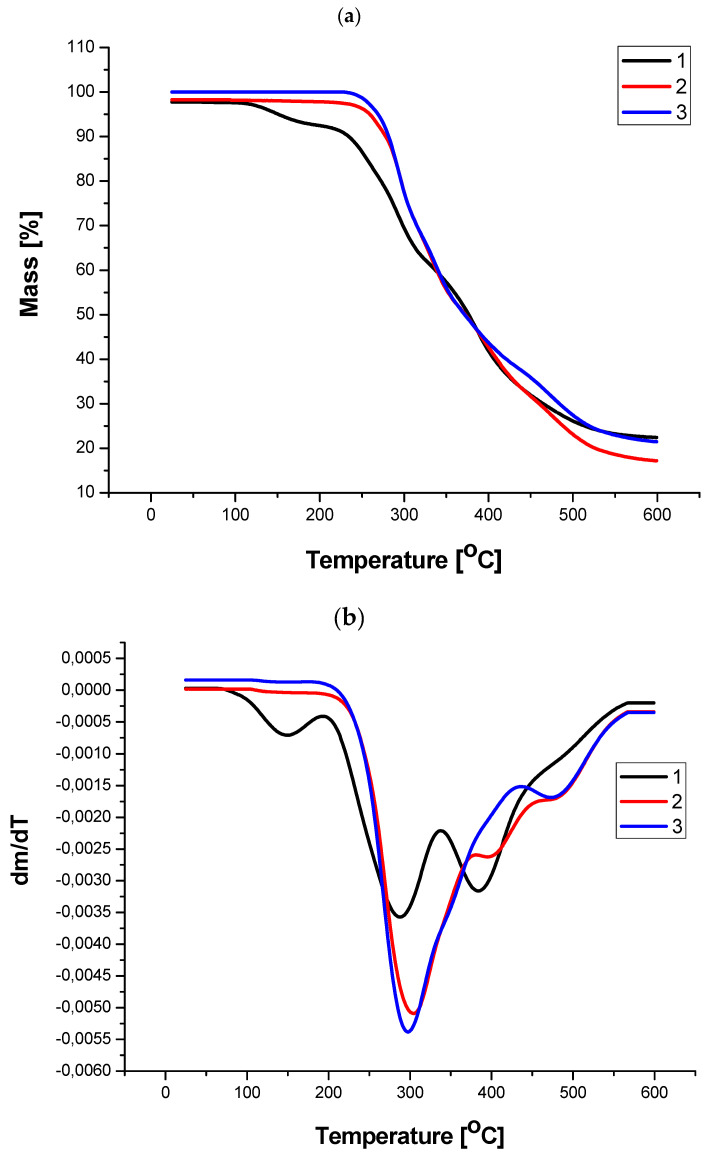
Thermal analysis of foams obtained from polyols H_3_PO_3_-DEG- HPC-GL-EC (1), HPC-GL-EG-EC (2), and HPC-TEG-GL-EC (3): mass change as a function of temperature (**a**); and differential mass change as a function of temperature (**b**).

**Figure 9:**
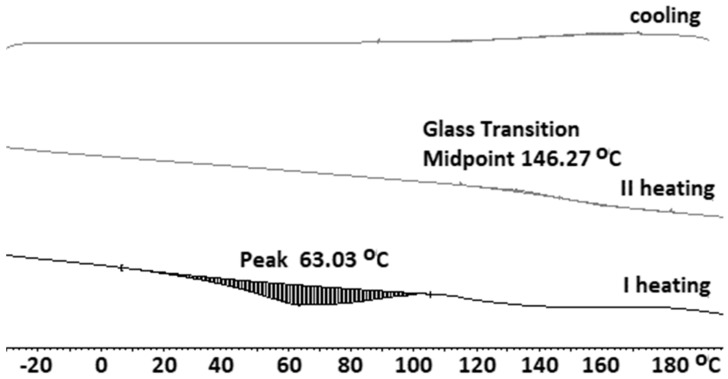
DSC thermogram of foam obtained from polyol H_3_PO_3_-GDE-HPC-GL-EC.

**Figure 10:**
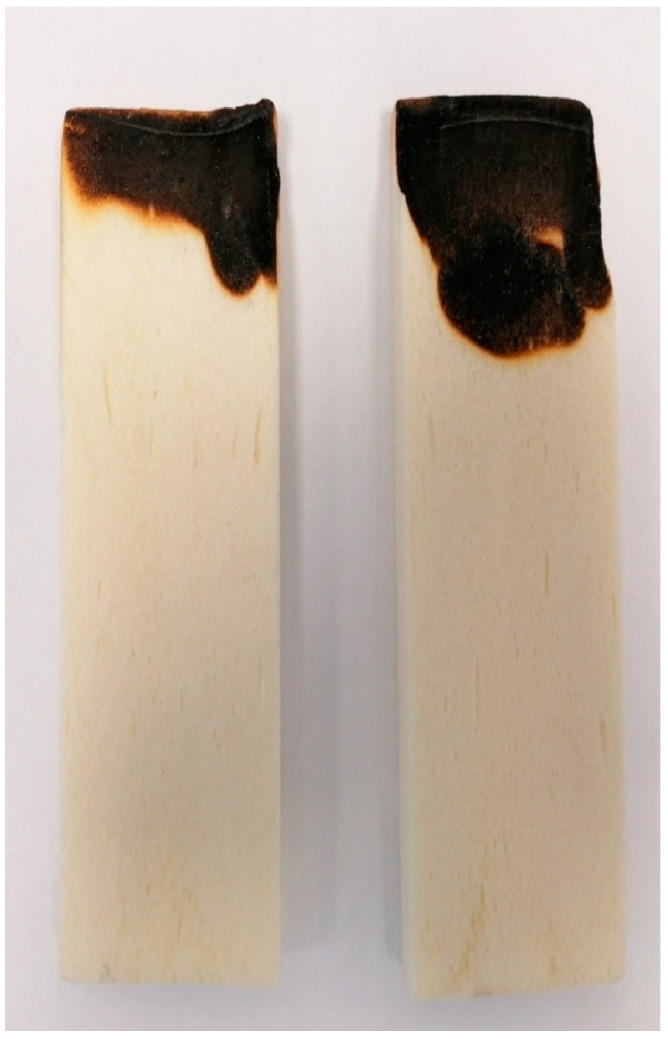
Photographs of PUF samples obtained from polyol H_3_PO_3_-DEG-HPC-GL-EC flamed at horizontal test.

Microscopic examination of PUF and its reference showed the oval pores in the materials (Figure 7, Table 6). The larger diameters are within 216–284 µm, while the smaller ones fall into the 99–155 µm region. The PUFs modified with flame retardants are more ellipsoid. This may be caused by a higher amount of TEA catalyst and shorter growth of PUF compared to a reference PUF. This is also responsible for thinner walls of pores in a modified PUF (Table 6). This explains why modified PUF has higher compressive strength (0.237 MPa) before thermal exposure than after thermal exposure to 175 °C (0.175 MPa). This is because thin pores crack easier (Table 5). The thermal resistance of obtained PUF was also analysed by dynamic method (Figure 8). The mass loss was observed already at 90 °C, i.e., lower than reference PUFs. We attributed this to the presence of a larger amount of TEA in the foaming process than in the case of unmodified PUFs. The boiling point of TEA is 101 °C. Fast decomposition occurs at 285 °C, again at a lower temperature than in reference foams (295–300 °C). The peak is related to the thermal decomposition of polyurethane into amine and carbon dioxide [29]. The third peak observed at 380 °C is due to the decomposition of ether bonds in polyols [30,31]. The DSC analysis allowed us to determine the glass transition temperature, which was 146 °C (Figure 9), that qualifies the obtained PUF as rigid PUF. Modified PUFs, as well as the reference PUFs obtained from HPC without flame retardants, were tested for flammability in the horizontal test (Table 7, Figure 10). It has been demonstrated that reference PUFs flamed out completely, with a rate nine times higher than that of modified PUFs.

The mass loss of modified PUFs was merely 5%, while it was 68% for reference PUFs. The oxygen index of PUF modified with phosphorus and melamine was above 21%, which was 2.7% higher compared with the reference PUFs. Another test related to flammability was performed using a cone calorimeter (Table 8). This method is considered the most reliable blazing test on a small scale [32]. The results obtained from the cone calorimeter enabled us to determine the following: TTI—the time to ignite; TTF—total time of flaming; PML—percentage mass loss; HRR—heat release rate; EHC—effective heat of combustion; and THR—total heat release.

**Figure 11:**
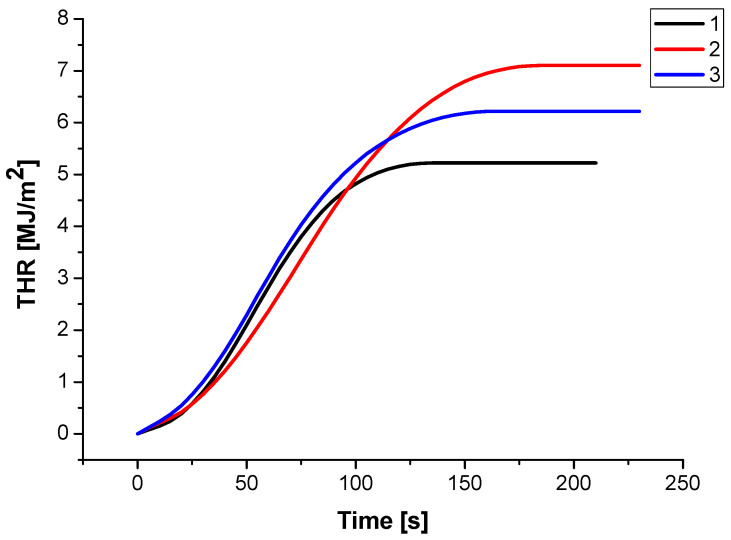
Total heat release vs. time during combustion of polyurethane foams obtained from polyols H_3_PO_3_-DEG-HPC-GL-EC (1), HPC-GL-EG-EC (2), and HPC-TEG-GL-EC (3).

**Figure 12:**
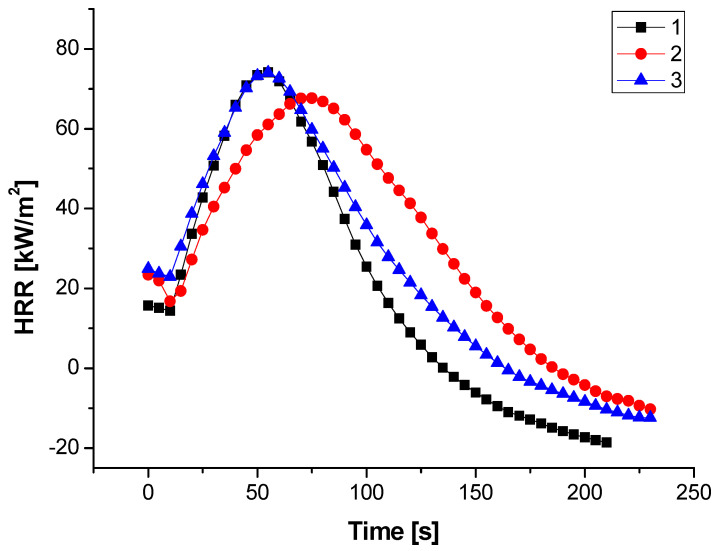
Heat release rate vs. time during combustion of polyurethane foams obtained from polyols H_3_PO_3_-DEG-HPC-GL-EC (1), HPC-GL-EG-EC (2), and HPC-TEG-GL-EC (3).

One of the parameters characterizing the heat resistance of materials is the time to ignite (TTI); the longer TTI means the longer time needed to heat up and ignite. The TTI for non-modified PUF was determined at 5 and 8 s. The flame retardant-modified PUF showed a TTI of 5 s, which was probably related to the higher apparent density of the foam. Thus, the values of TTI are short and characteristic of pored materials. Percentage mass loss (PML) for all samples is similar and does not depend on retardants. Effective heat of combustion (EHC) and total heat release (THR) of modified PUF are much lower in comparison with the reference PUF (Figure 11), which is an expected and demanded practical feature. The curves in Figure 12 illustrate the heat release time profile (HRR). The initial growth of the curve corresponds to the heating phase. At the ignition point, the maximum on the HRR curve is visible. Flame retardant-modified PUF (curve 1 in Figure 12) has a similar HRR to that obtained from HPC-TEG-GL-EC and reaches the maximum after 60 s, while that obtained from HPC-EG-GL-EC showed a maximum peak after 70 s and a smaller peak, which correlated with its lower apparent density. After reaching the maximum value on the HRR = f(t) curve, the heat relation rate decreases as a result of the reduction in the sample area covered by combustion. It is worth noticing that PUF modified both with phosphorus and melamine releases heat faster than the reference samples.

## 4. Summary and Conclusions

1. In the reaction between phosphoric(III) acid and diethylene glycol, the ester with terminal hydroxyl groups was obtained, which was further dissolved in HPC. It was hydroxyalkylated with glycidol and ethylene carbonate to give polyol with phosphorus atoms incorporated into the polyol;
2. The obtained polyol was reacted with polymeric diphenylmethane diisocyanate and water to obtain the rigid polyurethane foam. In the foaming process, melamine was also added as an additive flame retardant. The presence of incorporated phosphorus and added melamine led to the material of clearly reduced flammability, compared to foams based on HPC;
3. Obtained polyurethane foam had considerably higher thermal stability and lower polymerization shrink than other HPC-based polyurethane foams not modified with flame retardants. On the other hand, the obtained PUF had a higher apparent density, water uptake, and lower thermal resistance;
4. Foam modified with phosphorus atoms and melamine has a similar burning rate to unmodified foam obtained from HPC-based polyol in a diethylene glycol environment. Modified foam shows lower total heat release than non-modified one, which is its advantageous utility feature.

## Figures and Tables

**Table 1:** Comparison of the HPC elemental analysis determined with the calculated for subsequent hydroxypropyl cellulose derivatives.

Hydroxypropyl Derivative of Cellulose	%C	%H	%O
**HPC**	**53.21**	**8.44**	**38.35**
monosubstituted	49.09	7.27	43.64
disubstituted	51.80	7.91	40.29
trisubstituted	**53.57**	**8.33**	**38.10**

**Table 2:** Pre-foaming of polyol H_3_PO_3_-DEG-HPC-GL-EC.

Composition [g/100 g of Polyol]					Notes
pMDI	Water	TEA	Silicon L-6900	Melamine	
180	3	7.8	3.9	-	Slowly self-extinguishing
180	2	7.8	3.9	-	Slowly self-extinguishing
180	2	7.8	3.9	20	self-extinguishing

**Table 3:** The influence of composition on the foaming process of polyol H_3_PO_3_-GDE–HPC-GL-EC.

No	Composition [g/100 g of Polyol]					Isocyanate Index	Foaming Process			Notes
	pMDI	Water [%]	TEA	Silicon L-6900	Melamine		Cream Time [s]	Rise Time	Tack-Free Time [s]	
1	180	2	4.6	2.6	20	1.8	20	23	1	Viscous surface
2	180	2	5.8	2.6	20	1.8	15	10	1	Large pores
3	180	2	8.9	2.7	20	1.8	13	9	1	Small pores, fragile foam
4 *	180	2	7.8	2.7	20	1.8	18	9	1	Small pores, rigid foam

* Composition subjected to full characterization. Rise time—the time from the start of expansion to the moment of reaching the sample’s final volume. Tack-free time—the time from reaching the sample’s final volume to the moment of losing its surface adhesion to powdered substances. Tack-free time—immediately.

**Table 4:** Comparison of the properties of flame-retardant foam with reference foams without flame retardants.

Foam Obtained from Poyol	Density [kg/m^3^]	Absorption Of Water [% vol.]			Dimensional Stability of Foams [%] At 150 °C Temperature					
					Length Change After		Width Change After		Height Change After	
		After 5 min	After 3 h	After 24 h	20 h	40 h	20 h	40 h	20 h	40 h
H_3_PO_3_-DEG-HPC-GL-EC	74.4	1.67	2.50	4.79	−1.15	−1.22	−1.34	−1.76	−0.50	−1.64
HPC-GL-GE-EC	61.6	1.30	1.76	3.62	+3.51	+3.12	+6.72	+6.09	+1.19	+1.01
HPC-GL-TEG-EC	58.1	0.25	0.89	2.12	3.13	+4.56	−5.07	−4.16	−3.53	−2.89

Density was determined with ±3.4% accuracy. Standard deviation in case of absorption of water is 0.8%. Standard deviation in case of dimensional stability does not exceed 1.7%.

**Table 5:** Comparison of the thermal conductivity coefficient, mass loss, and compressive strength of flame-retardant foam with reference foams without flame retardants.

Foam Obtained from Poyol	Thermal Conductivity Coefficient [W/m·K]	Mass Loss [%] after Exposure to Temperature			Compressive Strength [MPa]			
					Before Exposure	After Exposure to Temperature		
		150 °C	175 °C	200 °C		150 °C	175 °C	200 °C
H_3_PO_3_-DEG-HPC-GL-EC	0.0361	19.0	31.7	-	0.237	0.350	0.173	-
HPC-GL-GE-EC	0.0358	12.4	28.9	45.3	0.475	0.499	0.533	0.606
HPC-GL-TEG-EC	0.0354	9.8	29.3	44.5	0.279	0.350	0.549	0.725

Heat conductance coefficient was determined with ±2.1% accuracy. Mass loss was determined with ±0.5% accuracy. Compressive strength was determined with ±2.8% accuracy.

**Table 6:** Results of micrograph analyses of foams.

Foam Obtained From Polyol	Larger Dia-Meter [μm]	Smaller Dia-Meter [µm]	Thickness of Cell Wall [µm]
H_3_PO_3_-DEG-HPC-GL-EC	234 ± 36	99 ± 20	8 ± 1
HPC-GL-EG-EC	216 ± 37	111 ± 36	9 ± 2
HPC-GL-TEG-EC	284 ± 57	155 ± 37	10 ± 2

**Table 7:** The results of the horizontal combustion test and oxygen index determinations of PUFs.

Foam Obtained From Polyol	Extent of Burning [mm]	Flame Rate [mm/s]	Mass Loss Upon Flaming [%]	Oxygen Index [%]
H_3_PO_3_-DEG-HPC-GL-EC	30–40	0.5	5.1	21.7
HPC-EG-GL-EC	150	4.3	68.4	19.0
HPC-TEG-GL-EC	150	4.4	67.5	19.0

**Table 8:** Flame retardance studies of PUFs measured by cone microcalorimeter.

Foam Obtained From Polyol	TTI [s]	TTF [s]	PML [%]	HRR [kW/m^2^]	THR [MJ/m^2^]	EHC [MJ/kg]
H_3_PO_3_-DEG-HPC-GL-EC	5	90	77.5	74.12	5.1	46.30
HPC-GL-EG-EC	8	158	79.5	67.67	7.1	64.29
HPC-GL-TEG-WE	5	149	82.5	74.03	6.2	60.32

## Data Availability

The original contributions presented in the study are included in the article.
